# mHealth Assessment: Conceptualization of a Global Framework

**DOI:** 10.2196/mhealth.7291

**Published:** 2017-05-02

**Authors:** Meghan Bradway, Carme Carrion, Bárbara Vallespin, Omid Saadatfard, Elisa Puigdomènech, Mireia Espallargues, Anna Kotzeva

**Affiliations:** ^1^ Norwegian Centre for E-health Research University Hospital of North Norway Tromsø Norway; ^2^ UiT The Arctic University of Norway Department of Clinical Medicine Tromsø Norway; ^3^ Universitat Oberta de Catalunya (UOC) Catalonia Spain; ^4^ TransLab Research Group Departament de Ciències Mèdiques Universitat de Girona (UdG) Catalonia Spain; ^5^ Red de Investigación en Servicios Sanitarios en Enfermedades Crónicas (REDISSEC) Bilbao Spain; ^6^ Mobile World Capital Barcelona Barcelona Spain; ^7^ Agència de Qualitat i Avaluació Sanitàries de Catalunya (AQuAS) Catalonia Spain; ^8^ CIBER de Epidemiología y salud Pública (CIBERESP) Madrid Spain

**Keywords:** mhealth, evaluation, assessment, checklist, framework

## Abstract

**Background:**

The mass availability and use of mobile health (mHealth) technologies offers the potential for these technologies to support or substitute medical advice. However, it is worrisome that most assessment initiatives are still not able to successfully evaluate all aspects of mHealth solutions. As a result, multiple strategies to assess mHealth solutions are being proposed by medical regulatory bodies and similar organizations.

**Objective:**

We aim to offer a collective description of a universally applicable description of mHealth assessment initiatives, given their current and, as we see it, potential impact. In doing so, we recommend a common foundation for the development or update of assessment initiatives by addressing the multistakeholder issues that mHealth technology adds to the traditional medical environment.

**Methods:**

Organized by the Mobile World Capital Barcelona Foundation, we represent a workgroup consisting of patient associations, developers, and health authority representatives, including medical practitioners, within Europe. Contributions from each group’s diverse competencies has allowed us to create an overview of the complex yet similar approaches to mHealth evaluation that are being developed today, including common gaps in concepts and perspectives. In response, we summarize commonalities of existing initiatives and exemplify additional characteristics that we believe will strengthen and unify these efforts.

**Results:**

As opposed to a universal standard or protocol in evaluating mHealth solutions, assessment frameworks should respect the needs and capacity of each medical system or country. Therefore, we expect that the medical system will specify the content, resources, and workflow of assessment protocols in order to ensure a sustainable plan for mHealth solutions within their respective countries.

**Conclusions:**

A common framework for all mHealth initiatives around the world will be useful in order to assess whatever mHealth solution is desirable in different areas, adapting it to the specifics of each context, to bridge the gap between health authorities, patients, and mHealth developers. We aim to foster a more trusting and collaborative environment to safeguard the well-being of patients and citizens while encouraging innovation of technology and policy.

## Introduction

Mobile health (mHealth) is defined by the World Health Organization’s (WHO) Global Observatory for eHealth as “medical and public health practice supported by mobile devices, such as mobile phones, patient monitoring devices, personal digital assistants (PDAs), and other wireless devices” [1]. Despite the lack of safety and quality validation of these technologies by medical regulatory bodies, individuals have adopted mHealth devices as self-management aids, while medical professionals are at a loss for how to relate to them [1]. Due to this consumer-based and rapid introduction within the world of patient health aids, mHealth solutions present unique and stakeholder-specific challenges to the medical environment. Patients, health care providers, administrators, authorities, and mHealth developers alike are operating without clear direction—from potentially improper use of mHealth apps by individuals, to medical systems’ inability to react due to lack of technological and organizational support. Therefore, several questions arise: How should health authorities approach mHealth evaluation and certification? How can this be accomplished without stifling innovation? Should efforts to determine risks, benefits, and appropriate use be held on a global, country, or regional basis? How will policies and strategies be introduced to medical professionals and their practices? Which methods should be used to ensure that patients are properly informed on how to select and use mHealth solutions?

Today, there are many efforts underway to address these challenges [2-5]. However, these are happening in silos and are often specific to a single country or medical system. In this paper, we explore the progress and setbacks of the mHealth assessment environment and propose a collaborative and global approach to assessment, with the aim of applying the competencies of all stakeholder groups. We believe that by structuring our work in this field from a common foundation, assessment initiatives can and will foster a more trusting and collaborative environment to safeguard the well-being of citizens while encouraging innovation of technology and policy.

The purpose of this viewpoint paper is to be informative and provocative, focusing on the changes in mindset and actions that must occur—from the education and perspective of consumers, health assessment evaluations teams, and medical authorities—in the assessment of mHealth solutions. As such, we hope to elicit discussion on the content and methodologies of our suggested common framework for mHealth assessment.

## The Potential of Mobile Technologies in the Medical Field

The potential of mHealth solutions lies in their ability to enable chronic diseases management and general wellness motivation [6-8]. By tracking an individual’s health and lifestyle data, and by providing actionable feedback, these tools encourage self-management. With the subsequent promise of equipping health care providers with such detailed information about a patient’s health status in relation to their daily habits, the medical system could not only better understand elusive compliance issues but also propose tailored solutions [9] for individual patients. If integrated and supported appropriately, these tools could improve treatment, empower patients, and foreseeably lower medical costs and streamline use of health care resources [10,11].

However, because these innovations are diverse and unstandardized, mHealth requires that regulatory bodies take a fresh look at evaluation methodologies for health aids. Today, medical actors are expected to adapt to the rapid technology turnover, ubiquity, and connectivity of mHealth by collecting, incorporating, and analyzing unprecedented amounts of personal health information seamlessly within the clinical environment. However, medical research and validation protocols, which providers rely on for guidance, are currently unable to adapt as quickly as such mHealth tools are being released. As a result, health care providers are left in the dark regarding how to relate to mHealth tools, as well as the impacts to their own professional responsibilities. If this goes un-checked, the medical system will continue to lag behind the needs of its patients.

## The Current Situation

Just as in the proverbial scenario of the Band-Aid on the leaking dam, the mHealth environment has been met with a series of incomplete or issue-specific solutions to the evaluation of health apps, wearables and sensors. In fact, neither the Health Technology Assessment (HTA) agency [12] nor its collaborative networks, European Network for Health Technology Assessment (EUnetHTA) and HTA Network, have tackled mHealth solutions within their range of technology assessments. As patients continue to use these apps regardless of clinical support or guidance, health authorities and providers fall further behind, stuck in the traditional and hypercontrolled operations of the medical sector. With good intentions, organizations continuously attempt to address the distinct issues facing patients and clinicians. Promising attempts included the National Health Service’s Health Apps Library [13], which aimed to involve clinicians in the review process, yet it is still under maintenance; the Organization for the Review of Care and Health Applications [14], which describes the purpose of their value and risk scores but not which features of an app lead to its value or risk; and PatientView’s “The myhealthapps directory 2015-2016” [15], which presented a summary of consumer-generated app reviews. While these initiatives attempt to provide the public with digestible and relevant information, they simply add to the slew of disjointed and static solutions that are not able to address all stakeholder issues.

### A Step in the Right Direction

Recently, governmental organizations and those representing end-user interests have acknowledged that flexible solutions and continuous communication are more appropriate for the mHealth environment than customary static reports. A preparatory review of these evaluation initiatives illustrates the range of developed mHealth evaluation methodologies and adapted health assessment frameworks for use in mHealth. These initiatives focus mainly on the usability and clinical application of mHealth technologies, which are most commonly provided by individual user's commentaries, the organization’s own evaluation team or, very rarely, a representative group of medical practitioners [16-19]. Some, including the United States Food and Drug Administration (FDA), choose to regulate only devices that fall under the definition of “medical device,” enabling them to rely on existing frameworks for evaluation and implementation [20]. Others attempt to address a broader range of tools, with some focusing only on risk assessment [21,22] or even utilizing nontraditional resources (eg, novel technologies and social media outlets [23]) to evaluate mHealth solutions. Finally, most initiatives address only mHealth solutions that are fully operational within the market [24], with no solution for those under development [25].

### Too Many Initiatives, Too Few Answers

Despite the diversity of approaches, recent reviews reveal worrisome results—a lack of conclusive or actionable evidence to suggest the ability of commercially available mHealth tools to effect behavioral changes or to manage chronic diseases, inpatient care, or health care delivery [26]. As a result, a primary concern and unfortunate reality is that this environment has allowed for the existence of misinformation regarding appropriate uses for these mHealth solutions. Common labels may even clearly categorize an app as “medical” yet include the warning in fine print that the app is “intended for entertainment only” [27]. Moreover, these evaluation efforts do not involve or inform all stakeholders [28] of relevant results, including potential risks associated with misleading health recommendations or software failures.

To compare the scope of structure and insights offered by today’s mHealth evaluation initiatives to the desired coverage and impact that we believe is possible, we have completed a comparative summary of representative evaluation efforts (see Multimedia Appendix 1 [29-33]). These characteristics are in addition to the assumed basic coverage of usability and security. Such a lack of flexible and inclusive evaluation options has resulted in a lack of empirically demonstrated understanding of the benefits and risks that mHealth solutions provide to care delivery and health management. There is clearly a need to create an environment that explores additional options for mHealth evaluation. For example, individuals outside the medical realm have not traditionally had a majority voice in the assessment of health aids. Yet today, individual app users are becoming more knowledgeable about the daily benefits of mHealth and technology developers are exploring the potential of ever-present self-management systems. Therefore, we should consider these groups as underutilized resources—we have an opportunity to recognize and enthusiastically encourage the insight that these individuals can now contribute to mHealth evaluation efforts.

### The Unsung Role of Developers in Assessment

Developers are the key force driving the need for certification due to the astounding rate at which they are producing mHealth solutions [34]. Therefore, those who are developing mHealth solutions should be encouraged to seek assessment for their own technologies through engagement in the evaluation process. Additionally, they should be provided with guidance and educational resources, including explanation of the concepts and benefits of a quality assured and reliable product go hand-in-hand with growth in the market and success, as the result of consumer engagement, loyalty, and trust [35,36]. By creating a mutually beneficial situation for developers and the medical system, evaluation frameworks can facilitate an environment that is transparent, trustworthy, and safe for users. It must also be noted that we must achieve a balance between development oversight and creative freedom. Too much regulation, or an unnecessarily lengthy process, would paralyze certification and inhibit adoption of mHealth solutions, which is evident from responses to the US FDA’s complex and unclear rulings [37]. Therefore, involving developers in the improvement and operation of any mHealth assessment initiatives will ensure the safety of their creativity and the competitive, open health-app market.

## The Need to Work From a Common Foundation

While the resources, evidence, and financial support for clinical implementation undoubtedly vary between countries and medical systems, there are shared challenges and themes for assessment. By acknowledging and addressing these common needs, we as a health care community can work in parallel as opposed to reinventing the wheel. Furthermore, these needs cannot hope to be addressed by a single organization or single perspective (eg, safety and privacy vs usability and consumer cost). The needs presented by the mHealth assessment arena can be met only through participation of all stakeholders, hand-in-hand with regulation and legislation, constantly adapting to updates in standards and the capacity of technology.

To facilitate the success of this collaborative approach, we must simultaneously change the paradigm of evaluation to more appropriately relate to the particularities of mHealth. As such, it must be continuous and iterative, while at the same time provide timely conclusions and actionable recommendations for improvement and implementation.

## Acknowledging and Merging Universal Needs: Approaching the Solution Together

Organized by the Mobile World Capital Barcelona Foundation, the co-authors of this paper represent a workgroup of several international partners with distinct yet complementary backgrounds and competencies related to mHealth, including patient groups, government, and health authorities, as well as representatives from health research, care providers, and technology developer groups. Exploration of the common needs and themes of mHealth has resulted in a summative list of concepts that we believe are universal to all assessment initiatives. We welcome interested parties and future partners to contribute their competencies and applicable solutions toward the future development of common and foundational guidelines for building mHealth assessment frameworks. Not only are newly validated tools needed in order to improve the quality of assessment steps, but also the perspectives of a diverse group of representatives in all affected fields. The following sections outline the major universal concepts and approaches that can inform proposed and existing evaluation initiatives.

### Assigning the Evaluation Team

The concept of specialization is a key component in any effort to impact a multistakeholder system. A single organization cannot be expected to accomplish the diversity of tasks and successfully address the challenges of mHealth evaluation (eg, results of safety, usability, and health change assessments). Therefore, representative organizations should be involved in tasks associated with their competencies, thus providing even distribution of responsibilities as well as relevant input.

In order to achieve a comprehensive process, the evaluation team should possess a broad scope of perspectives that are representative of the following stakeholder groups: patients or patient organizations; commercial and research-based mHealth developers; health care providers, medical professionals, and system administrators; insurance or other reimbursement bodies; and authorities within governmental health and medical system organizations.

It is assumed that the composition of this team, and specific roles of stakeholder representatives, will vary based on the decision of each country or region that chooses to adopt and adapt this approach to their own respective medical system.

### Pre-Assessment of mHealth Solutions

**Figure 1:**
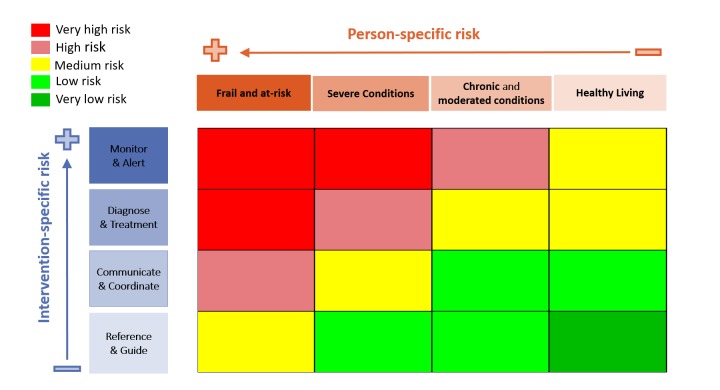
Risk assessment matrix.

The pre-assessment phase is meant to classify any mHealth app and can be based on, for example, the following criteria: (1) Risk classification matrix: combining intervention type and patient type (see Figure 1 [38] for an example); (2) Users: patients, medical professionals, and/or informal caregivers; and (3) Integration: stand-alone, partially integrated, or fully integrated.

In order to address mHealth solutions within any stage of development, pre-assessment guides should be distributed to developers who are still in the process of designing health solutions as well as for members of evaluation teams within any medical system or country.

### Checklists

Together with the pre-assessment phase, checklists can act as preparative resources for the evaluation teams to ensure that all relevant information is provided before resources and time are spent assessing an mHealth solution, as well as subsequent public and medical integration. Once pre-assessment has been established, checklists can be directed toward each contextual category of health apps. The checklist will vary from country to country and be provided to the evaluation team. To illustrate this point, a checklist should ensure that documentation related to the following categories is provided for each mHealth solution:

- designation of mHealth solutions by intended use, for example, reference guides (eg, for nutrition or weight control), monitoring devices (eg, for blood sugars or blood pressure), or other types of solutions within a matrix
- level of development
- security and privacy
- interoperability standards
- usability
- functionalities and content

To the best of our knowledge, there are few functional and representative checklists (eg, Catalonia, Andalucía, and WHO’s mHealth Technical Evidence Review Group [29,39,40]). Most others are not fully available, are under development (eg, European Commission mHealth assessment working group [5]), or are focused only on mobile medical devices (eg, Future Internet-STAR checklist model [41]). These checklists propose categories related to functionality including usability, technology, security, content design and pertinence, and services. They also propose more domains related to infrastructure, including intervention delivery, accessibility toward individuals (ie, barriers or facilitators to the adoption of the intervention among study participants), cost assessment, adoption inputs/program entry, limitations for delivery at scale, contextual adaptability, and replicability. Many of these approaches involve classification of information into levels, from mandatory to not applicable, based on the app’s level of risk, thereby providing flexibility to assess not only medical but also nonmedical devices.

Again, each stakeholder representative within evaluation teams should be assigned to the topics they are most capable of assessing. However, not all of these available checklists are accessible or usable by all stakeholders. As previously mentioned, developers are a key stakeholder in the success of assessment initiatives. Therefore, all developers should be able to provide the checklist information themselves. An alternative and more time-consuming option is that facilitators be put in place to gather this information from multiple sources in order to answer evaluation questions. This will affect the level of time and funded investment needed, which supports the involvement and support of developer groups in the design of checklists.

### mHealth Evaluation Aspects and Methods

Assessment initiatives must be equally focused on summative (ie, during or post implementation) as well as formative (throughout the development life cycle) evaluation. This will ensure that not only common software development life-cycle stages but also end-user needs and input are incorporated, thus giving any initiative practical dimension. We expect that in doing so, this will promote adoption by the app development community as well as those representing the interests of the medical community.

The categories of information provided by checklists can be evaluated with the common evaluation perspectives of technical readiness and maturity, risks, benefits, and resources needed. The limitations and specifics of what is involved in these evaluation domains should correspond to the purpose of the evaluation (ie, assessing a device as an educational or medical tool) and should vary depending on the level of interoperability and intended use (eg, disease self-management vs activity tracking) of the mHealth solution (eg, stand-alone app vs integrated medical device).

Each of these four domains and their subdomains should be defined to streamline evaluation efforts and organize stakeholder participation based on their respective competencies (see Multimedia Appendix 2 for an example). Ideally, the chosen set of domains and subdomains should address the following needs:

- Determine the appropriate use of each mHealth solution (ie, as a medical device or a health and wellness tool, based on the target and breadth of functionalities as well as status of interoperability and safety standards)
- Develop expedited and conclusive methods to evaluate the effect(s) that an mHealth solution has on respective clinical outcomes and/or patient lifestyle habits, based on its appropriate and intended use
- Assess risk related to (1) patients and their caregivers in relation to personal data security, self-management decision making, and disease understanding; (2) clinicians, including liability to their practice and a greater trust of and reliance on patient-gathered data; and (3) overall health care organizations and systems, including financial impact and liabilities
- Inform stakeholders of relevant results through respective and accessible platforms.

Quantitative or qualitative methodologies may be employed through formal evaluation studies (eg, clinical trials) to assess each of these. The evaluation studies should be conducted based on the regulations, practices, and implementation strategies of individual countries or medical systems in which such studies are completed. However, to our knowledge, most of the subdomains still lack validated, standardized, or descriptive approaches and methods for evaluation. Therefore, alternative or unconventional inquiry methods should be considered including involvement of medical education programs as well as clinical and commercial research organizations, all of which use complementary methods such as online platforms, market analysis, and user-involved workshops. Partnership with organizations that are able to develop such novel methods should be sought both within the international realm of health sciences and then adapted to the needs and socioeconomic factors present within each unique medical system.

## Conclusions

There is a clear need for defining a standard assessment framework for mHealth technologies that will help separate the wheat from the chaff and identify those solutions that may provide added value to patients and the health care system. By accomplishing this set of methodologies and approaches to evaluation and also the methods, perspectives, and resources used to accomplish these tasks, evaluation would facilitate more educated and informed decision making regarding the choice and use of mHealth solutions—from patients to medical practitioners to the health authorities that are charged with maintaining the foundation of each medical service.

The insights and suggestions provided in this paper are intended for the groups that are completing evaluations, developing apps, and creating health policy and infrastructural support for mHealth implementation. We propose that all organizations and individuals who share a similar passion for a coordinated effort towards a more rigorous and comprehensive evaluation of mHealth technologies join forces to form a virtual community of practice [42]. Discussing the merits and shortcomings of the proposed approach in this paper and its utility in real-world scenarios can be a starting point for such community. We hope that a free-flowing format will foster creative ways to solve the problems put forward in this position paper. A social media‒based discussion group is a feasible starting point for this virtual community of practice. The authors invite the experts in evaluation of health information systems to provide their opinion as to how such a virtual community should form and collaborate.

## Appendix

Multimedia Appendix 1 Presence of desirable characteristics within existing mHealth assessment initiatives.

Multimedia Appendix 2 Example set of domains and subdomains for an mHealth assessment framework.

## Abbreviations

EUnetHTA: European Network for Health Technology Assessment
FDA: Food and Drug Administration
FI-STAR: Future Internet Social and Technological Alignment Research
HTA: Health Technology Assessment Agency
mHealth: mobile health
WHO: World Health Organization
