# Supplementary material for: mHealth Assessment: Conceptualization of a Global Framework
Source: JMIR Mhealth Uhealth. 2017 May 2;5(5):e60. doi: 10.2196/mhealth.7291 (PMC5434253; doi:10.2196/mhealth.7291)
Supplement: Multimedia Appendix 1 [file mhealth_v5i5e60_app1.pdf]

	**EC**[39]	**Catalonia**[40]	**Andalucía**[35]	**FI-STAR**[42,43]
The model is applicable to all mHealth solutions, as opposed to merely medical devices	Y	Y	Y	N
Stakeholders from multiple fields were involved in the creation of the model	P	Y	Y	N
Stakeholders from multiple fields are given roles within the model for practical assessment of apps	P	Y	P	N
The results of assessment are/will be publically available and easily accessible, i.e. clear and understandable lay language	Y	Y	Y	N
The model assess the credibility of the developer as well as the app, given its intended use and assigned risk level	N	Y	Y	NS
The model adapts to the level of app development, i.e. from developmental to observational testing to apps already on the market	N	Y	N	Y
The model addresses the concept of potential app-integration within the medical system	Y	P	NS	Y
The model equally covers risks or perspectives of patient as well as medical personnel users	Y	P	P	NS
The model assesses an app's infrastructure, given its intended use and risk, e.g. potential interoperability, support services, user-guides	Y	P	P	Y
The model includes references to information and security standards as resources for evaluation teams	Y	N	N	Y
The model is applicable to more than one country or regional medical system	Y	NS	Y	Y
The model allows for use of novel evaluation methods to expedite the collection and reporting of results	NS	NS	NS	N
