# Supplementary material for: mHealth Assessment: Conceptualization of a Global Framework
Source: JMIR Mhealth Uhealth. 2017 May 2;5(5):e60. doi: 10.2196/mhealth.7291 (PMC5434253; doi:10.2196/mhealth.7291)
Supplement: Multimedia Appendix 2 [file mhealth_v5i5e60_app2.pdf]

	User	Organization	Healthcare System	Society
**Technical Readiness & Maturity**	• Usability<br>• User experience<br>• User-centred design<br>• Suitability<br>• Reliability	• User support<br>• Compliance with interoperability standards<br>• Clinical Integration (IT and protocols)<br>• Implementation reliability	• Compliance (interoperability standards, certifications)<br>• Technical validity and precision<br>• Reliability of measures<br>• Replicability	• Legal compliance (data protection)<br>• Regulatory compliance (if medical device)
**Risks**	• Technical (security, privacy, electricity-related)<br>• Clinical and behavioural<br>• Inadequate contents (lack of updates, evidence-based functions or information)<br>• Economic (inc. costs, time)<br>• Ethical (stigma due to visibility of devices)	• Technical (data-related: leak, loss, error; electricity-related)<br>• Decrease in quality of care (not integrated or in contradiction with operating pathways, protocols)<br>• Economical (increased costs if the solution is not cost-effective, or due to overuse of services)	• Decrease in quality of care (ineffective solutions)<br>• Economical (increased costs due to technical or clinical cost, overuse of services)<br>• Inequality of access (if cost for the end user is too high)	• Inequality of access<br>• Unintended consequences (environmental changes)<br>• Over-diagnosis
**Benefits**	• Health<br>• Behavioural change (lifestyles, self-management)<br>• Empowerment and satisfaction<br>• Improved access to care (waiting lists)<br>• Economical (cost and time savings)	• Process and workflow improvements<br>• Quality improvements (higher case resolution, decreased hospitalization rates)<br>• Economical (cost and time savings, budgetary impact)<br>• User satisfaction	• Improved quality of coordinate care<br>• Economical (cost and time savings, cost-effectiveness)<br>• Improved general health (decreased disease prevalence and incidence, QALYs gained)	• Decrease prevalence, increase awareness<br>• Increased productivity (decreased disability rates, lost work days)<br>• Economical (cost and time savings)<br>• Improved research focus<br>• Enhanced collaboration through information sharing
**Resources needed**	• Technical literacy<br>• Training<br>• Cost paid by the user (or financial support structure)	• Training provision<br>• Logistics<br>• Initial investment costs (devices, infrastructure, network)	• Training provision<br>• Initial investment costs (devices, infrastructure, network)	• Medical culture changes
